# The Role of Core Biopsy versus Vacuum-Assisted Breast Biopsy In Primary Breast Angiosarcoma

**DOI:** 10.1155/2021/9305811

**Published:** 2021-07-28

**Authors:** Anna Abate, Giulia Querques, Riccardo Giovanazzi, Camillo Di Bella, Valeria Besostri, Mara Gisabella, Cesare Maino, Davide Ippolito, Rocco Corso

**Affiliations:** ^1^Department of Radiology, San Gerardo Hospital, Monza, Italy; ^2^Department of Breast Surgery, San Gerardo Hospital, Monza, Italy; ^3^Department of Pathology, San Gerardo Hospital, Monza, Italy

## Abstract

We report the case of a 45-year-old woman with a slow-growing palpable nodule on the left breast, confirmed as a well-defined opacity on mammography, corresponding to a 5 cm hyperechoic lesion on ultrasound, and considered, on the basis of clinical examination and radiological findings, to be consistent with a lipoma. One year later, the patient represented with an enlarged left breast mass and underwent further imaging investigation with subsequent diagnosis of primary breast angiosarcoma obtained via a Vacuum-Assisted Breast Biopsy. The patient developed metastatic disease and succumbed to the disease one year after definitive diagnosis. Primary breast angiosarcoma is a rare malignant vascular neoplasia, characterized by aggressive patterns, poor prognosis, and absence of pathognomonic radiological features. Currently, there are no evidence-based guidelines regarding treatment, even though wide surgical resection followed by chemo- and radiotherapy appears to improve survival.

## 1. Case Report

A 45-year-old woman, with no history of breast cancer or chest radiotherapy, presented to an external institution for evaluation of a slow-growing palpable nodule in the left breast. Mammogram demonstrated a well-defined opacity with a corresponding 5 cm hyperechoic lesion on ultrasound (US), which was considered, on the basis of clinical examination and radiological findings, consistent with a lipoma.

**Figure 1:**
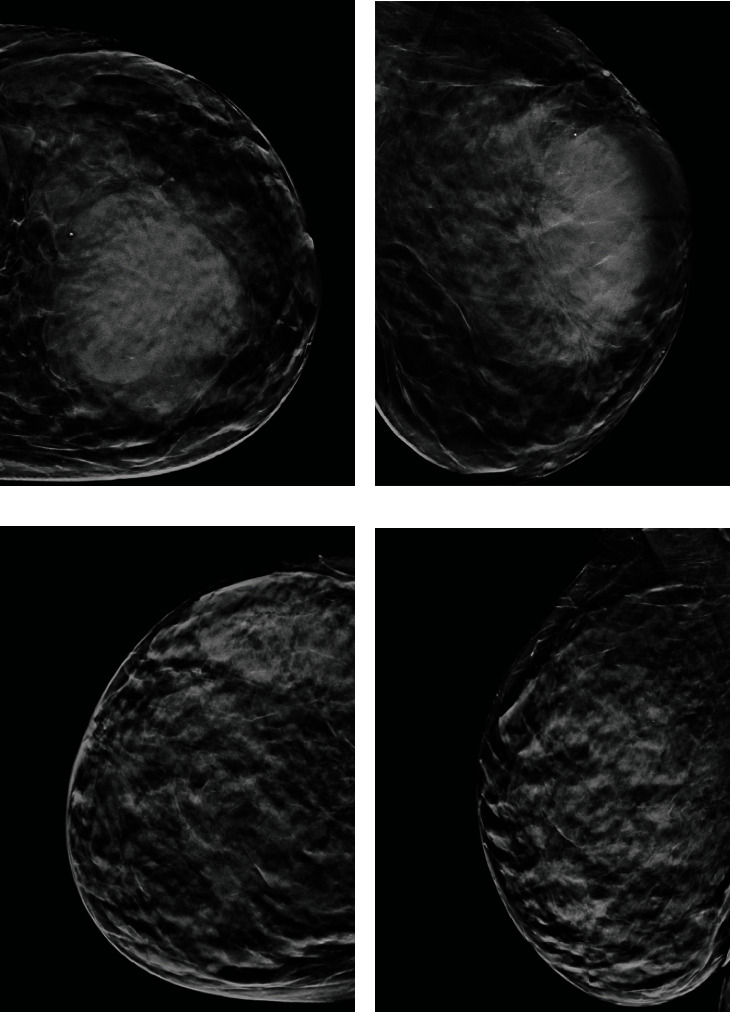


One year later, she presented to our department, with the same lesion in the left breast. On clinical examination, it appeared as a hard, fixed, and painless mass. Mammograms (Figure 1) demonstrated asymmetric breast enlargement with a large heterogeneous mass, compared to the right breast. US showed complete structural parenchymal distortion caused by an inhomogeneous and confluent 10 cm lesion, with increased color Doppler vascular signal associated with multiple large anechoic fluid areas.

A first free-hand core biopsy with a 14-G needle was performed, resulting in a histology report of necrosis and fibrin-leukocyte tissue (B1). Therefore, a second free-hand core biopsy was performed, and histology reported the presence of necrosis without any evidence of epithelial cells (B2). Subsequently, the patient developed a cutaneous fistula. Indeed, after two weeks, the multidisciplinary meeting decided to get another histopathologic correlation through a third core biopsy under US guidance with a 14-G needle resulting in a pathology report of necrotic and fibrin-hemorrhagic material with fibro adipose tissue fragments and small foci of atypical vascular proliferation (B4, CD31+/-, CD34+, FLY1+, Factor VIII-/+, MIB1 20%). Finally, a Vacuum-Assisted Breast Biopsy (VABB) with a large core 8-G needle (Mammotome Devicor Medical Products) was performed to in an attempt to further sample both the tumor core and perilesional parenchyma.

The histopathology of the vacuum biopsy resulted in large necrotic and hemorrhagic areas with atypical vascular proliferation (B5d, CD34+, ERG+, FLI+, Factor VIII+) and voluminous, hyperchromic, and irregular nuclei (Ki67 50%), suggestive of malignant vascular neoplasia consistent with primary breast angiosarcoma.

**Figure 2:**
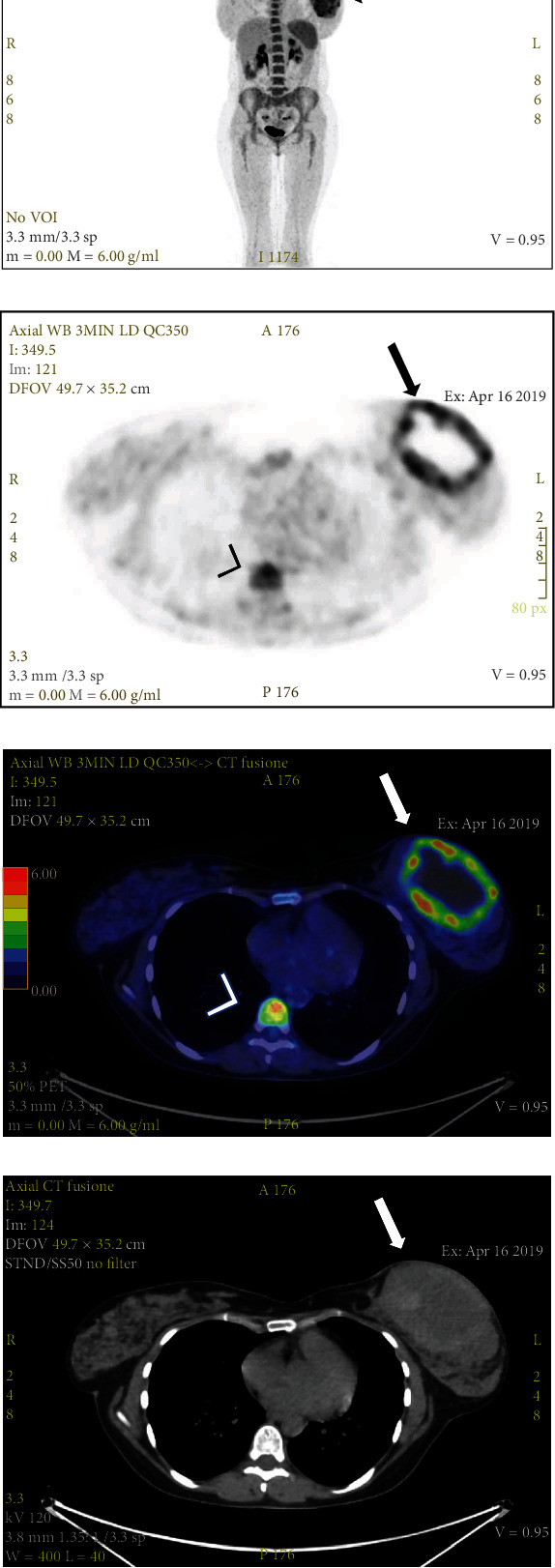


The patient underwent a combined fluorodeoxyglucose positron emission tomography-contrast-enhanced computed tomography (CT) (FDG-PET-CT), which demonstrated the presence of a tumoral mass with a central necrotic core in the left breast. This resulted in an inhomogeneous FDG uptake on PET images (Figures 2(a)–2(c)) (SUVmax 6.88), corresponding to an area of peripheral enhancement with a central hypoattenuation on contrast-enhanced CT (Figure 2(d)), respectively. Moreover, PET images allowed the detection of metastasis, with FDG uptake in a 1 cm nodule in the lower outer quadrant of the right breast with (SUVmax 3.15), not identified on mammography or US, and also in the vertebral body of T10 (SUVmax 6.25).

**Figure 3:**
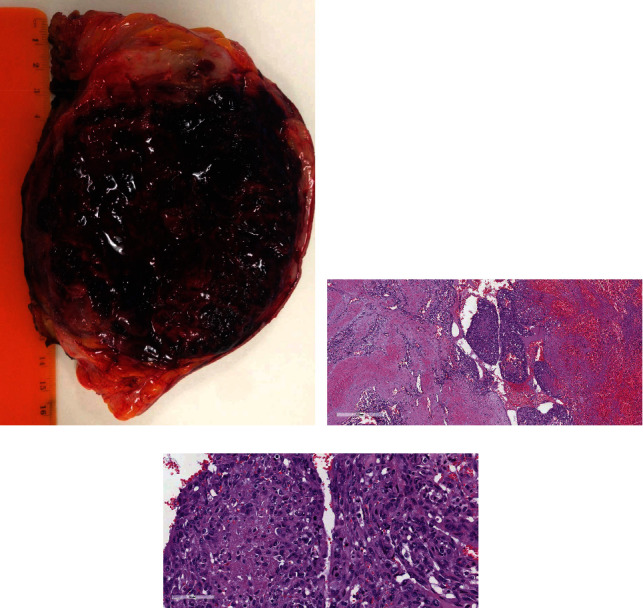


The patient was unable to undergo breast MRI due to the presence of bilateral cochlear implants and opted to undergo bilateral mastectomy. Final pathology reported wide ulceration of the skin and a greyish lesion with irregular margins and large areas of hemorrhagic cavitation (Figure 3(a)): the lesion was classified as a moderately differentiated (G2) primary breast angiosarcoma with solid components and necrosis (G3) associated with diffuse acute inflammation. The immunohistochemical investigation confirmed the vascular nature of the lesion (CD31, Factor VIII), with absent perineural invasion. Up to 25 mitoses for 10 HPF were observed, and staining for Ki67 highlighted the solid spindle cell areas of increased proliferation (Figures 3(b) and 3(c)). Histological analysis of the right breast revealed the presence of a metastatic lesion. After surgery, the patient underwent adjuvant chemotherapy with Gemcitabine and Taxotere. Follow-up included PET-CT examinations every two months. The patient subsequently developed further metastasis to the brain, lung, and bone and succumbed to the disease one year after definitive diagnosis.

## 2. Discussion

Breast angiosarcoma represents a rare malignant vascular neoplasm originating from endothelial cells, often high grade and multicentric, that can be easily misdiagnosed. It may occur in a primary form with onset in premenopausal age or a secondary form with onset in postmenopausal age typically in patients with a history of previous breast cancer treated with radiation therapy [1, 2].

Clinically, primary breast angiosarcoma presents itself as a palpable, rapidly growing mass, associated or not with subcutaneous edema which can be difficult to distinguish from radiation-induced thickening and, in some cases, with bluish skin discoloration due to the presence of vessels within the lesion [3, 4].

The diagnosis can be challenging given the nonspecific radiological signs; in particular, mammograms may show breast asymmetry or a poorly defined mass without spiculations or calcifications [4].

Indeed, in a study of 21 cases from Liberman et al. [3], 33% of the cases did not present with clearly recognizable mammographic features, while in a study by Yang et al. [4], 19% of patients with confirmed primary breast angiosarcoma had a mammogram score of BIRADS 2. At US, primary breast angiosarcoma may present as a well-circumscribed or ill-defined, solitary or multiple, focal or diffuse lesion, without posterior acoustic attenuation, associated with hypo-/hyper- or mixed echogenicity, and hypervascularization at color Doppler [3, 5, 6]. Furthermore, the presence of adipose tissue within the lesion can complicate the differential diagnosis with hemangiomas and angiolipomas. Primary breast angiosarcoma is better identifiable with MRI, appearing as an extended multilobulated mass, usually characterized by hyperintensity on T2-weighted sequences and inhomogeneous signal on T1-weighted imaging due to hypointense tumoral mass with hyperintense areas corresponding to hemorrhage and blood lakes, with prolonged enhancement after contrast media injection [6, 7].

Considering the difficulty of identifying primary breast angiosarcoma through imaging, the histopathological framework is of utmost importance. In this context, core biopsy with a standard needle (14-G) represents a faster, less invasive, less expensive, and more available procedure, without the risk of scarring or architectural distortion on subsequent mammograms, compared to VABB [8–11]. However, despite its high sensitivity (85–97%) and low false negative rate (2–7%), core biopsy is associated to significant underestimation of malignant breast lesions providing insufficient tissue or fragmented specimens, often being inconclusive due to sampling of necrotic tissue, and resulting in the need for further procedure in order to examine the entire lesion. Noteworthy, multiple grades may exist in the same tumor, and therefore, grading from core biopsy may not be feasible or be inaccurate. Moreover, there is no means of dealing procedural bleeding aside from manual compression.

On the other hand, VABB with a larger core needle (8-G) is less readily available and more expensive and time consuming, requiring higher expertise. Nevertheless, it allows a more complete sampling of the target lesion (about fivefold more material per core), resulting in a lower rate of histological underestimation and rebiopsy and lower false-negative results with an overall improvement in lesion characterization [12–14]. Moreover, due to the double aspiration system, it allows better control of bleeding through blood removal from the bioptic cavity. Finally, the single insertion of the needle compared to the repeated introduction during a standard core biopsy reduces the risk of fistulization when the lesion is localized near the cutaneous surface, as reported in our patient [15]. Of note, in our case, fistulization occurred after free-hand core biopsy which did not allow the visualization of the necrotic liquid areas, compared to biopsy performed under US guidance.

At macroscopic examination, primary breast angiosarcoma appears as a mass deeply located in the breast parenchyma with a spongy-hemorrhagic appearance and ill-defined margins. Poorly differentiated lesions can appear as solid fibrous nodules. Histologically, primary breast angiosarcoma is assigned a degree of differentiation according to the classification proposed by Donnell et al. [1]. Low-grade primary breast angiosarcoma shows similarities with benign vascular lesions such as hemangioma, angiomatosis, and pseudo-angiomatous stromal hyperplasia (PASH), which however have small dimensions and are well-defined without infiltrating growth patterns. Indeed, the main features that allow the correct identification of primary breast angiosarcoma are the infiltration of the surrounding tissues and the nuclear atypia of endothelial cells. For high-grade neoplasms, sarcomatoid carcinoma and other high-grade sarcomas should be considered for the differential diagnosis. Prognosis depends on tumor grade with 5-year survival varying between 75% for medium- to low-grade and 15% for high-grade forms with a recurrence rate of 45-65% [3, 16]. Prognostic factors include tumor size, resection margins, and patient's age. Metastases to regional nodes are rarely observed, as primary breast angiosarcoma more commonly spreads to the lungs, liver, and bones. Treatment is based upon histopathological features and includes surgery and adjuvant chemotherapy to reduce whole-body dissemination and local recurrence [16, 17].

In conclusion, in patients with suspected primary angiosarcoma, early detection and patient referral to high-volume dedicated centers are key factors to optimize patients' management. This is due to the availability of second-line biopsy such as VABB, a reliable and well-tolerated procedure, with higher diagnostic performance compared with core biopsy, due to better tissue sampling allowing for more accurate diagnosis with minimal associated complications.

## Data Availability

The data used to support the findings of this case report are available and included within the article.
